# Bayesian Source Attribution of *Salmonella* Typhimurium Isolates From Human Patients and Farm Animals in England and Wales

**DOI:** 10.3389/fmicb.2021.579888

**Published:** 2021-01-28

**Authors:** Mark Arnold, Richard Piers Smith, Yue Tang, Jaromir Guzinski, Liljana Petrovska

**Affiliations:** ^1^Department of Epidemiological Sciences, Animal and Plant Health Agency (APHA), Addlestone, United Kingdom; ^2^Department of Bacteriology, Animal and Plant Health Agency (APHA), Addlestone, United Kingdom

**Keywords:** source attribution, *Salmonella* Typhimurium, Bayesian modelling, SNP distance, multi locus sequence typing, core-genome multi locus sequence typing

## Abstract

The purpose of the study was to apply a Bayesian source attribution model to England and Wales based data on *Salmonella* Typhimurium (ST) and monophasic variants (MST), using different subtyping approaches based on sequence data. The data consisted of laboratory confirmed human cases and mainly livestock samples collected from surveillance or monitoring schemes. Three different subtyping methods were used, 7-loci Multi-Locus Sequence Typing (MLST), Core-genome MLST, and Single Nucleotide Polymorphism distance, with the impact of varying the genetic distance over which isolates would be grouped together being varied for the latter two approaches. A Bayesian frequency matching method, known as the modified Hald method, was applied to the data from each of the subtyping approaches. Pigs were found to be the main contributor to human infection for ST/MST, with approximately 60% of human cases attributed to them, followed by other mammals (mostly horses) and cattle. It was found that the use of different clustering methods based on sequence data had minimal impact on the estimates of source attribution. However, there was an impact of genetic distance over which isolates were grouped: grouping isolates which were relatively closely related increased uncertainty but tended to have a better model fit.

## Introduction

*Salmonella* is a common cause of foodborne illness in people, with over 90,000 cases reported in Europe annually (ECDC, 2016). Salmonellosis is typically associated with symptoms of diarrhoea, fever, and abdominal cramps and is linked to eating contaminated food. *Salmonella* is the second most common cause of gastrointestinal illness in the United Kingdom (UK), after *Campylobacter*. The number of United Kingdom laboratory reports of *Salmonella* in people in 2016 was 8,630 (PHE, 2018). However, a prospective community study determined that the true level of *Salmonella* infection in the United Kingdom could be five times greater in the community (Tam et al., 2012b). The main serotypes of *Salmonella* detected in people are *Salmonella* Enteritidis (SE) and *Salmonella* Typhimurium (ST), and these accounted for ∼50% of all non-typhoidal cases in England and Wales in 2016 (PHE, 2018). SE cases are largely linked to contact or consumption of poultry or eggs, whereas ST is present in a wide range of animal species and so it is more difficult to determine the source of ST cases.

The number of laboratory reports of *Salmonella* cases reduced from 12,094 in 2007 to 8,630 in 2016 (PHE, 2018). This was supported by the results of community cohort studies in 1993–1996 and 2008–2009 which showed that the number of *Salmonella* cases had reduced between these two periods from 0.8 to 0.3% (Tam et al., 2012a). The decrease was largely down to the reduction in cases infected with SE, which reduced from 6,489 to 2,356 during this period, whereas the number of cases with ST had remained largely stable with ∼1,700 cases each year on average. It is largely believed that this reduction was due to the introduction of *Salmonella* control plans within the poultry industry, including the use of vaccination, which reduced the number of human cases related to poultry.

In the late 1990s, monophasic variants of ST (*S*. 4,5,12:i- and *S*. 4,12:i-), with the absence of one or both flagellar antigens (second phase H antigens), were detected in animals and have become increasingly prevalent, with a subsequent rise in human cases being infected with these monophasic variants (EFSA, 2012). Monophasic ST (MST) had been detected in the United Kingdom in the early to mid-1990s but these were likely to have been non-typable strains, rather than true monophasic variants, lacking the flagellin B gene (Mueller-Doblies et al., 2018). In the United Kingdom, veterinary surveillance data indicated that MST was detected in various livestock species from 2006 to 2013 (Petrovska et al., 2016) but typically at a low overall prevalence, whereas the prevalence in pigs has been increasing since 2009. The results from an abattoir study detected a prevalence of *Salmonella* of 30.5% in caecal content samples collected from slaughtered pigs, with ∼34.4% of isolates reported as monophasic variants of ST and 19% as biphasic ST (Powell et al., 2016).

Although reported prevalence of *Salmonella spp*. varies among European Member States, pork meat is implicated as the highest salmonellosis foodborne infection risk to human health (Food Control Consultants Ltd Consortium [FCC], 2011). Other sources from the food production chain contribute to a varying degree to the human infections with ST and MST, and travel is also considered as an important “source” of sporadic salmonellosis. Surveillance of *Salmonella* from United Kingdom primary production of food animals is necessary for gaining knowledge on the most important sources or reservoirs of *Salmonella*, as well as the principal routes of transmission.

*Salmonella* Typhimurium and MST have more than one animal host reservoir and even though ST is a closely related group, they contain variants that exhibit distinct phenotypes associated with virulence and epidemiology. Source attribution approaches to quantifying the importance of different sources and transmission routes amongst animals, direct animal transmission or through the food chain to humans, are important for policy makers to understand where to target interventions, and also for prevention of outbreaks and sporadic foodborne infections. One common approach to source attribution is the frequency-matching methods, which infer the overall source attribution by comparing the relative frequency of animal isolates of the same subtype as human isolates. A source attribution method based on frequency matching that has been used in many countries both in Europe (Hald et al., 2007; Mughini-Gras et al., 2014) and elsewhere (Mullner et al., 2009; Glass et al., 2016) is a Bayesian approach first developed for Denmark (Hald et al., 2004). Subtyping of isolates for source attribution has usually been carried out using phenotypic data, such as serotype or phagetype. However, the additional data generated by the increasing use of whole genome sequencing (WGS) of bacterial pathogens leverages WGS variation data to track genotypes in their host populations and into the food chain. This enables the relating of important genotypic differences to phenotypes associated with foodborne pathogens, and thus may allow for a development of more accurate source attribution models.

Due to the emergence of MST in animals and people, it is important to ascertain the most likely animal sources of ST and MST cases in the United Kingdom. It is hoped that with the increased specificity provided by WGS sequencing techniques, it will be easier to highlight which sources are a risk to human health. The aim of this study was to apply the modified version of the Hald model (Mullner et al., 2009) to United Kingdom data from England and Wales, where subtyping was carried out using sequence data.

## Materials and Methods

**FIGURE 1:**
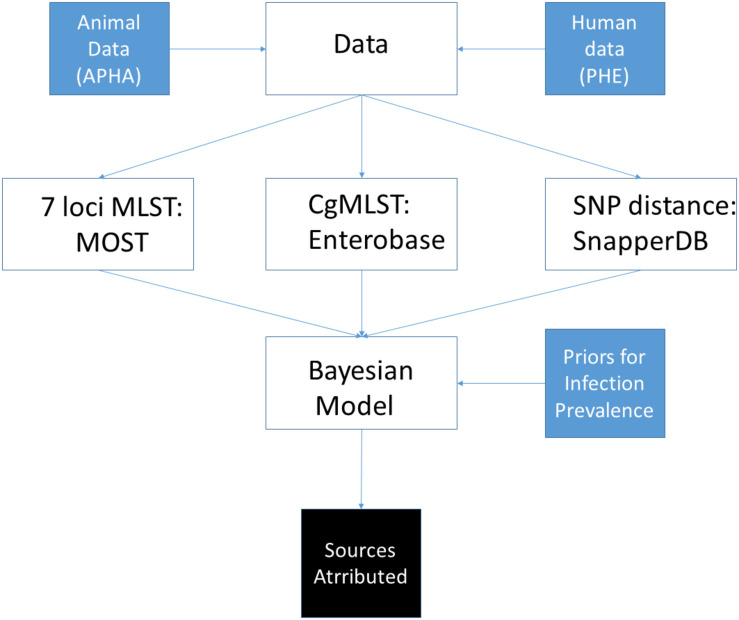
Overview of steps undertaken to perform source attribution using sequence data from *S*. Typhimurium isolates from England and Wales.

An overview of the main activities involved in data collection, processing and source attribution modelling is given in Figure 1, with details given for each of the individual steps below.

### Sequenced Population

The selection criteria for inclusion and minimum required animal and human sample sizes was defined by the COMPARE project^1^ benchmarking study workgroup (Munck et al., 2020a). A set of sequenced isolates of ST and MST strains from 596 human and 327 animal sources were collected from England and Wales. The animal isolates were available through national surveillance/monitoring/control programs/research projects conducted between 2014 and 2016. The set criteria by the COMPARE project were to include, where possible, a minimum sample size of 25 isolates per animal species, per country, per year. Laboratory isolations of *Salmonella* from British livestock are reportable under the Zoonoses Order (1989) and samples are either sent to Animal and Plant Health Agency (APHA) laboratories or isolations must be reported to APHA. However, only a subset of these *Salmonella* isolates are sent for sequencing, typically in outbreaks or for research purposes, with only a small population randomly selected each year for sequencing.

All sequenced human ST and MST isolates of human cases from England and Wales during the years 2014–2016 were evaluated in the study. The information on the identifiers of the sequenced isolates available in the public domain was obtained from Public Health England (PHE). The sequence data of 596 human isolates was downloaded from EBI^2^ and serotyped *in silico* using SeqSero (Zhang et al., 2015), to confirm the serotype.

To reduce the chances of human to human spread being the source, non-index cases that were defined as part of an outbreak by PHE were omitted. Additionally, cases that had reported foreign travel prior to illness were also omitted from the dataset.

### DNA Extraction, Whole Genome Sequencing and Quality Control

Overnight cells of the selected animal isolates were pelleted by centrifugation and re-suspended in 0.5 mL 0.1 M PBS (pH7.2) solution. Genomic DNA was extracted using the ArchivePure DNA Cell/Tissue (1 g) kit (5 Prime, Gaithersburg, United States). Extracted genomic DNA was fragmented, tagged for multiplexing with the Nextera XT DNA Sample Preparation Kit (Illumina, United Kingdom) and sequenced at the APHA on the Illumina MiSeq platform using 150 base paired-end reads according to the manufacturer’s instructions. Quality control and filtering of the sequenced 327 animal and 596 human genomes was performed at the Computerome facility at the DTU^3^ using the FoodQC pipeline (Zhang et al., 2015). Briefly, BBDuk program^4^ was used to remove the contaminating Illumina adapters and to filter out bases with the PHRED score below 20. Only reads that were longer than 50 bases after these operations were retained (if just one of a pair of reads was under 50 bases, the other read in the pair was also removed). FastQC 0.11.5^5^ was used to visually assess the quality of reads before and after trimming. The retained reads were *de novo* assembled using SPAdes 3.11.0 (Bankevich et al., 2012), and the quality of the assemblies was assessed in Quast 4.5. (Gurevich et al., 2013). An isolate was retained if its assembly comprised less than 500 contigs larger than 500 bases, was associated with an N50 value above 30,000, assembly size was approximately 5 million bases, and the average coverage across the genomes was higher than 30×.

### Sequence Typing by cgMLST, HierCC, 7-loci MLST, and SNP Distance–Preparation of Inputs for the Bayesian Source Attribution Models

The core genome Multi-Locus Sequence Typing (cgMLST) profiles were generated at the DTU utilizing the 3002 locus Salmonella Enterobase cgMLST scheme (Alikhan et al., 2018) in Bionumerics version 7.6 (Applied Maths, Sint-Martens-Latem, Belgium). A further filtering step was implemented at this stage to exclude isolates with more than 5% missing data resulting from too low genomic coverage at the cgMLST loci (i.e., no reliably assigned alleles at more than 2852 out of 3002 core genome loci). Subsequently all retained human and animal isolates were grouped into core genome sequence types using Enterobase^6^ such that all isolates with identical allele profiles across all 3002 cgMLST loci were assigned the same sequence type. A hierarchical clustering algorithm in Enterobase^7^ was then applied on the sequence types to cluster isolates into four disparate Hierarchical clustering classes (HCC) at 10, 20 50, and 100 cgMLST allele differences representing most and least genetically homogenous isolates, respectively. The sequence types based on 7-loci MLST scheme were assigned to all isolates using MOST (Tewolde et al., 2016). SnapperDB software (Dallman et al., 2018) was used to assign a single nucleotide polymorphism (SNP) address to each selected human and animal isolate based on their SNP distance using *Salmonella* Typhimurium LT2 (AE006468) as reference. The “genetic distance” (number of SNP differences) is calculated between all pairs of isolates within the specific eBG. Hierarchical grouping of isolates into clusters based on increasing levels of similarity is performed by calculating the pairwise SNP distance for each pair of isolates in the analysis set and performing a single linkage clustering at 250, 100, 50, 25, 10, 5, and 0 SNPs from the deduced distance matrix (SNP address). The resultant “SNP address” provides an isolate level nomenclature where two isolates with the same SNP addresses have 0 SNP differences (Achtman et al., 2012). The range of SNP/CgMLST differences considered for the subsequent source attribution analysis was restricted to those ranges between 10 and 100, as it was considered that differences of 5 or less would be too discriminatory and ranges over 100 would link together a high proportion of isolates that were not genuinely epidemiologically linked.

### Bayesian Model

The analysis was based on the Hald model of source attribution (Hald et al., 2004), with the modifications suggested in a previous study (Mullner et al., 2009), allowing for the use of typed sequence data. Briefly, the model uses the relative frequency of occurrence of *Salmonella* subtypes in the animal and human case data to infer the proportion of human cases that derive from each animal source. It is assumed that human cases from source *i* and subtype *j*, *O*_*ij*_, follow a Poisson distribution with mean λ_*ij*_, which was given by:

$$\lambda_{i\,j}=p_{i\,j}\,q_{i}\,a_{j}$$

where *p*_*ij*_ represents the prevalence of type *i* in food type *j*, *q*_*i*_ represents the relative virulence of each bacterial subtype, and *a*_*j*_ represents the relative likelihood of infection for each food source. The prevalence *p*_*ij*_ was itself given by the product of two factors, π_*j*_, the prevalence in food source *j* and *r*_*ij*_, the relative prevalence of type *j* in source *i*.

Each *p*_*ij*_ was estimated following the approach described in a previous study (Mullner et al., 2009), for the scenario where only positive cases were subtyped. For this, beta distributions for each *p*_*ij*_ were derived from the observed number of positive samples of each type and each food source, assuming a vague prior for the prevalence in each food source (π_*j*_ ∼beta(1,1)) and a vague Dirichlet prior for each *r*_*ij*_.

Priors for both *q*_*i*_ and *a*_*j*_ followed that suggested in Mullner et al. (2009). Prior distributions for *q*_*i*_ of bacterial strain type parameters were modelled hierarchically, with log(*q*_*i*_)∼*N*(0,τ), so that the overall mean of the *q*_*i*_ would equal 1 but with variation between subtypes given by *τ*, which itself had a relatively uninformative prior (gamma with both parameters equal to 0.01. Each *a*_*j*_ followed an exponential (0.002) prior, which was fairly uninformative but prevented very large values of the *a*_*j*_.

The Bayesian source attribution model depended on the infection prevalence in each of the animal sources. Data availability for estimation of infection prevalence in the animal sources varied between animal types (Table 1), and given the uncertainty in the prevalence estimates, results were initially produced assuming vague priors for the prevalence for all animal species (using beta distributions for the prevalences with both parameters set equal to 1, which is uniform between 0 and 1). However, to determine the impact of this assumption on the source attribution, informative priors were obtained where possible and the model re-run using these. The most robust data source was for pigs, where a Bayesian model was used to infer prevalence from a recent abattoir survey where three tests were applied in parallel to slaughter data (Powell et al., 2016). Infection prevalence for poultry (layers, broilers, and turkeys) was estimated using data from *Salmonella* National Control Programmes, taking into account the sensitivity of the tests (Arnold et al., 2009; Arnold et al., 2014). Estimates for cattle and sheep were taken from data on the proportion of positive *Salmonella* submissions to surveillance, and an estimate of the sensitivity of this surveillance by comparing the proportion of positive samples from *Salmonella* submissions and an abattoir prevalence survey for *Salmonella* in cattle and sheep in 2003, and taking into account the sensitivity of the test (Arnold et al., 2015). For game birds and pets it was felt that insufficient data was available to inform a prior for infection prevalence and so these were kept as vague priors.

**TABLE 1:** Priors used for animal infection prevalence for a Bayesian source attribution model for *Salmonella* Typhimurium (including monophasic variants) in Great Britain.

Animal source	Prior prevalence (%) (95% CrI)	Beta prior	Source
Broilers	0.31 (0.19–0.30)	Beta(14,4143)	Data from NCP in GB
Cattle	0.38 (0.03–0.57)	Beta(18, 4431)	Abattoir survey (Milnes et al., 2008) and APHA surveillance reports (APHA, 2014, 2015, 2016)
Game birds	Non-informative	Beta(1,1)	
Layers	0.21 (0.11–0.48)	Beta(22, 3698)	Data from NCP in GB
Other mammals	Non-informative	Beta(1,1)	
Pigs	27.4 (21.0–51.7)		Abattoir survey (Powell et al., 2016)
Sheep	0.12 (0.05–0.27)	Beta(7, 5582)	Abattoir survey (Milnes et al., 2008) and APHA surveillance reports (APHA, 2014, 2015, 2016)
Turkey	0.38 (0.17–0.38)	Beta(7, 1676)	Data from NCP in GB

*NCP, National Control Programme; GB, Great Britain.*

All calculations were performed in WinBUGS 1.4, using a burn-in of 5,000 iterations followed by 20,000 iterations of the model. To assess convergence, the model runs were performed several times with different starting values followed by inspection of the history of each parameter and the use of the Gelman-Rubin statistic.

The fit of each model to the data was assessed by the use of Bayesian *p*-values, which are a measure of model fit based on Pearson chi-square statistics (Gelman et al., 2003), where a low *p*-value represents a poor fit of the model to the data. The WinBUGS code for the model was adapted from a previously supplied code (Nerette et al., 2008).

## Results

### Summary of Sequenced Isolates Included in Analysis

To create a population of sequenced isolates from animals that could represent the major food animal reservoirs and thus reflect what humans are exposed to either through consumption of food or direct exposure to infected animals, genomes of 327 isolates from seven different groups of animal species were evaluated for inclusion in the study. The animal food sources included in the model were: chickens (broilers and layers), cattle, game birds, turkeys, pigs, and sheep, and an “other mammals” category was also included, which represented human exposure through contact with animals, such as horses and other companion animals. A total of 280 animal isolates selected for the study period reached the selected sequence data quality threshold (85.6% of all sequenced animal isolates). These included 163 isolate from pigs (58.2%), 42 from other mammals (15.0%), 20 from cattle (7.1%), 18 from game birds (6.4%), 14 from turkeys (5%), 9 from broilers (3.2%), 7 from sheep (2.5%), and also 7 from commercial egg-laying flocks (Supplementary Table 1). A total of 109 were ST strains and 171 were MST variants. Comparisons to the proportion of all English and Welsh surveillance isolates suggests that the sequenced dataset was generally representative of isolates collected during that period, although there were fewer cattle isolates and more from game birds (APHA, 2014, 2015, 2016; Table 2). The resulting total of animal isolates included in the analysis, by species and *Salmonella* type (whether ST or MST), is shown in Supplementary Table 1.

**TABLE 2:** Comparison of the distribution of *Salmonella* Typhimurium and monophasic variants in different in animal sources from routine surveillance and those selected for whole genome sequencing.

Animal species/Salmonella type	Salmonella isolations reported from routine surveillance				% of animal type from overall total	% in sequenced dataset
	2014	2015	2016	Total		
Cattle				95	14.1%	7.1%
Stm	25	17	20			
4,5,12:i-	8	6	6			
4,12:i-	5	4	4			
Sheep				17	2.5%	2.5%
Stm	3	0	10			
4,5,12:i-	0	1	0			
4,12:i-	0	2	1			
Pigs				387	57.5%	58.2%
Stm	75	42	43			
4,5,12:i-	36	39	47			
4,12:i-	26	49	30			
Chickens				33	4.9%	5.7%
Stm	2	6	7			
4,5,12:i-	10	0	1			
4,12:i-	2	3	2			
Turkeys				38	5.6%	5.0%
Stm	1	2	2			
4,5,12:i-	17	8	4			
4,12:i-	3	0	1			
Horses				84	12.5%	15.0%*
Stm	21	17	17			
4,5,12:i-	12	5	5			
4,12:i-	3	3	1			
Game birds				19	2.8%	6.4%#
Stm	6	3	2			
4,5,12:i-	0	0	0			
4,12:i-	0	0	8			

Stm = *Salmonella* Typhimurium. *Figure relates to all Other Mammals. #Figure relates to all game birds.

The human dataset included 596 genomes of which 294 (49.3%) reached the selected sequence data quality threshold for the study. A further 21 (7.1%) were omitted as they were found not to have been sampled in the years of interest (2014–2016), 47 (16.0%) had reported foreign travel, 44 (15.0%) were non-index cases that were part of an outbreak, 5 (1.7%) had reported foreign travel and were also part of an outbreak. The remaining 177 isolates were selected for use in the source attribution analysis and included 83 ST strains and 94 MST variants.

### Summary of Results From WGS Based Typing Methods

Three different WGS based typing methods were applied to establish the genetic relatedness amongst the sequenced human and animal isolates, 7-loci MLST, cgMLST and SNP address. Of the different typing approaches, 7-loci MLST had the fewest number of subtypes and had the greatest number of animal isolates that were of the same subtype as a human isolate (i.e., the sum of the human cases attributable to an animal source) (Table 3). Both SNP distance and HCC approaches had more human isolates attributable to animal sources isolates as the clustering/SNP distance thresholds for isolates to be grouped together increased. However, increasing the SNP distance threshold had a much larger impact on the number of human and animal isolates that were of the same subtype than increasing the HCC threshold. HCC consistently grouped more isolates than SNP distance, especially for low distance thresholds. The number of attributable subtypes (i.e., the number of subtypes that had both animal and human isolates) did not vary greatly between the SNP distance and hierarchical clustering approaches, but there were fewer for 7-loci MLST, it having only two attributable sequence types.

**TABLE 3:** Number of subtypes and animal samples of the same subtype as a human isolate for subtyping by SNP distance (SNP), 7 loci MLST, and hierarchical clustering (HCC) of core-genome MLST data.

Subtyping method/distance	Total number of subtypes	Number of attributable subtypes	Number human samples attributable (out of 177)	Percent human samples attributable
SNP10	231	8	19	10.7
SNP25	99	7	88	49.7
SNP50	67	8	104	58.8
SNP100	47	7	108	61
MLST	10	3	172	97.2
HCC10	122	9	91	51.4
HCC20	68	8	125	70.6
HCC50	45	9	139	78.5
HCC100	28	11	155	87.6

### Results From Bayesian Source Attribution Model

**FIGURE 2:**
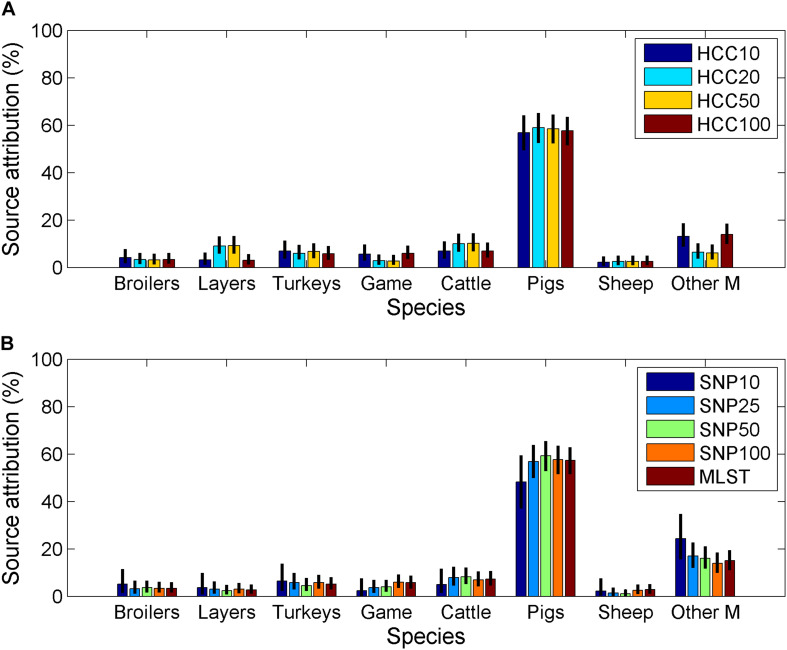
Estimated source attribution for *Salmonella* Typhimurium (including monophasic variants) for **(A)** isolates typed using core-genome MLST data and **(B)** isolates typed by SNP distance or 7-loci MLST (Other M = other mammals, Game = game birds).

The largest contributor to human infection from ST (including MST) was pigs, which was likely to have caused the majority of human cases (Table 4 and Figure 2). Other mammals and cattle were the next largest contributors. Game birds and sheep were estimated to contribute the least to human infection.

**TABLE 4:** Source attribution (%) and model fit (in terms of Bayesian *p*-value) for England and Wales from *Salmonella* Typhimurium and monophasic variants, estimated from a Bayesian model applied to three different methods of strain typing.

Source	Source attribution by clustering method and distance (%)								
	SNP				7-loci MLST	HCC			
	10	25	50	100		10	20	50	100
Broilers	5.0	3.2	3.6	3.4	3.4	4.1	3.3	3.2	3.4
Layers	3.7	3.1	2.4	3.0	2.7	3.1	9.1	9.2	3.0
Turkey	6.5	5.8	4.5	5.8	5.1	7.0	6.0	6.7	5.8
Game birds	2.3	3.6	4.0	6.0	5.8	5.7	2.8	2.7	6.0
Cattle	5.0	7.9	8.3	7.0	7.3	6.9	10.0	10.2	7.0
Pigs	48.2	56.9	59.3	57.7	57.3	56.9	58.9	58.5	57.7
Sheep	2.3	1.4	1.1	2.5	2.8	2.2	2.6	2.6	2.5
Other mammals	24.2	17.0	16.1	13.9	15.0	13.2	6.5	6.2	13.9
Bayesian *p*-value	0.13	0.06	0.08	0.09	0.06	0.16	0.09	0.13	0.09

While there were no major differences between source attribution estimates for the different clustering methods/distances, there were some differences in the findings. The source attribution estimate for game birds varied from 2.3% (SNP distance of 10 to group isolates) to 6.0% (SNP, HCC distance of 100 to group isolates) (Table 4). Source attribution estimates for sheep tended to be higher using the hierarchical clustering data (core genome MLST) than 7-loci MLST or SNP distance. Other mammals had a higher estimate of source attribution for SNP10 than any other clustering approach. Increasing the SNP distance to group isolates tended to increase the source attribution estimate for pigs slightly, although there was no corresponding pattern for HCC as the clustering distance increased.

**FIGURE 3:**
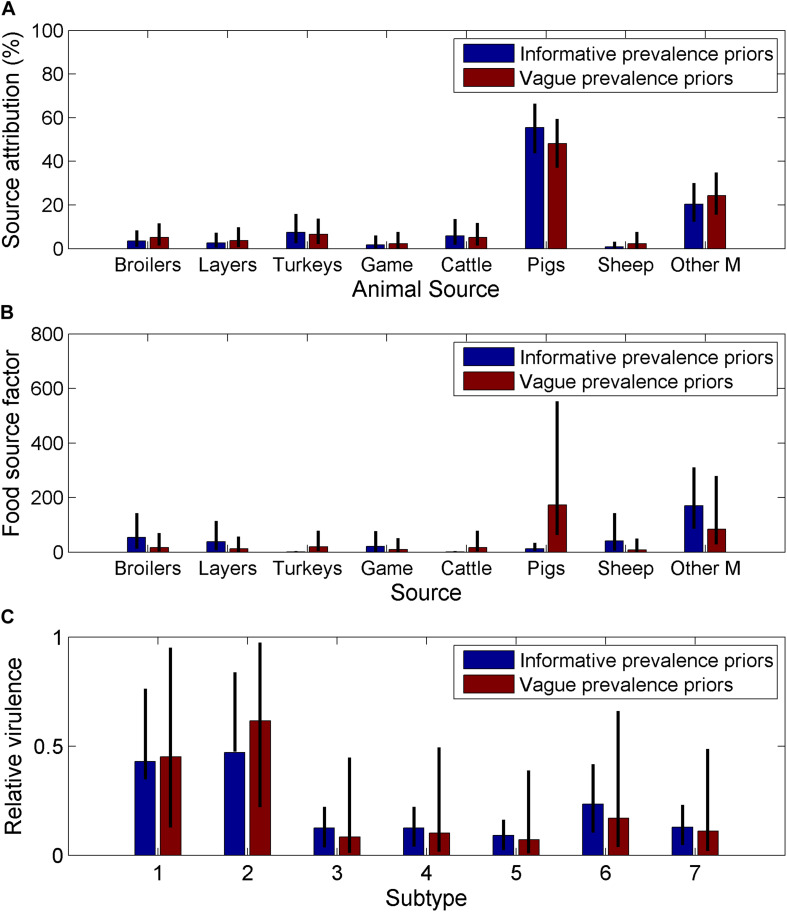
Comparison of estimates from a Bayesian source attribution model for England and Wales when using informative and non-informative priors on **(A)** the source attribution estimates for each animal source **(B)** animal source dependent factors, which is a summary measure of the ability of the source to act as a vehicle for human infection, and will depend on human consumption/contact with the animal source, and the environment provided for the bacteria through storage/preparation and **(C)** relative virulence by subtype, which is a measure of the relative ability of the subtype to cause human disease based on a combination of pathogenicity, survivability and virulence (Other M = other mammals, Game = game birds), the seven different SNP10 types were arbitrarily labelled 1–7.

The use of informative priors for the animal prevalence only had a small effect on the source attribution estimates (Figure 3A) or the subtype virulence estimates (Figure 3C) but had a large impact on the animal source factors (Figure 3B). The animal source factor looks at whether the source attribution is in line with or differs from what would be expected according to the relative infection prevalence in the source. Therefore, the changes in the animal source factors reflects the relative difference between the informative and vague prior for infection prevalence, with pigs having the highest prevalence for the informative prior, this had the greatest impact when amended to a vague prior.

In terms of model fit (using the Bayesian *p*-value), all subtyping methods resulted in a *p*-value of >0.05 (Table 4), so there was no strong evidence of a lack of fit of the models to the data. The greatest *p*-values (suggesting the better fit) resulted from HCC10 and SNP10 i.e., the subtyping methods with the greatest discriminatory power. While MLST, which had the least discriminatory power, had the joint equal lowest Bayesian *p*-value, there was no clear pattern of discriminatory power versus model fit for the other subtyping methods. The HCC typing approach tended to have higher *p*-values than its SNP equivalent for the same genetic distance, suggesting it to be a slightly superior approach based on model fit alone. As an additional check, correlation between parameter estimates were estimated and found to be low for most pairwise parameters combinations (Supplementary Figure 1, Supplementary Data).

## Discussion

The present study has used subtyping methods based on sequence data to cluster isolates and then apply a frequency based Bayesian model to infer source attribution, known as the Hald model. The advantage of the Hald model is that is a transparent, well established approach that can be used in tandem with more complex methods to provide comparison and validation. It also provides additional estimates, i.e., source specific and subtype specific relative risks, along with the source attribution. The disadvantage of the approach is that it can only infer source attribution for the animal subtypes where there is a corresponding human case of that subtype. Alternative approaches to source attribution which attempt to allocate a source for all the human isolates have recently been developed based on machine learning (Lupolova et al., 2017; Munck et al., 2020a,b) or on multinomial logistic regression (Guillier et al., 2020). These methods attempt to determine host specific features of the core genome or accessory genes that can then be used to infer the source of each human isolate. Another approach recently applied to ST/MST isolates from Denmark (Merlotti et al., 2020), involves creating a network where the isolates are the nodes and isolates are considered linked if their genetic distance is below a specified threshold, the idea being that isolates from the same source will cluster together. This approach has some elements in common with the Bayesian approach applied in this study, in that in both approaches a threshold measure of genetic distance is applied in order to subtype or cluster the isolates, although the attribution calculation methods differ. It would be useful to apply these different approaches together with the modified Hald approach to make a comparison of the performance of each method; this is the subject of a current research study. In particular, one feature of the Bayesian approach that is not replicated in the alternative, recently developed methods, is that the Bayesian approach allows the use of informative priors, which is particularly useful in representing the infection prevalence in the different sources. The alternative methods implicitly assume that the training data set represents the epidemiology of *Salmonella* in the sources included, and it would be useful to see whether this impacts the source attribution estimates.

The main contributors to human ST/MST infection were found to be pigs in this study, by far the greatest source, followed by other mammals and cattle. A study looking at source attribution for ST/MST in Denmark similarly found pigs to be the greatest contributor, with 53% of human cases estimated to be from Danish pigs, and 16% from imported pigs (Munck et al., 2020b). However, this finding in the present study is dependent on the representativeness of the source data used as inputs to the source attribution model. The initial target of having 25 samples per animal source was not met as there were very few sequenced isolates of ST/MST in some of the sources, and the large estimate of the source attribution for ST/MST from pigs was driven by the large number of samples from pigs. The Hald method depends upon both the infection prevalence of ST/MST in the animal source population and the distribution of subtypes within that source in the population. Where there are few samples for a particular source, then it is possible that the distribution of subtypes is poorly characterized, creating the possibility that its attribution is underestimated in the model. This means that there is uncertainty in the estimates of source attribution derived in the present study due to data limitations. In theory, sequencing all the samples from a particular animal source should avoid the lack of representativeness, but in practice only a proportion of the infected animals will be detected by surveillance, especially where animals are asymptomatic. This is a difficult problem to currently overcome, and will affect all source attribution methods based on microbial subtyping. In the present study we tried to improve representativeness by sampling over 3 years to increase the number of samples per animal source, and further sampling and sequencing in future years should increase the sequenced isolates per animal source and assist representativeness, as long as there are no temporal trends in the subtype distribution in the population.

The source attribution estimates from the present study were found to be similar for the subtyping method and the various genetic distances used to cluster isolates. On this basis, one might suggest that those subtyping methods which had the greatest number of animal isolates of the same type as human isolates would be the favoured approach, as this leads to more animal isolates contributing to the final estimates and consequently reduced uncertainty (Figure 1). However there is a risk that the subtyping methods that group together isolates that are potentially genetically distant, especially 7-loci MLST, 100SNP, and 100HCC, could in some cases lead to misleading results. Hence there was a trade off in varying the genetic distance over which isolates were subtyped. Increasing the genetic distance led to more animal source isolates being of the same type as a human isolate, thus reducing uncertainty in the source attribution estimates (Figure 1). Reducing the genetic distance, led to greater certainty that subtypes contain genuinely related isolates, but led to more human subtypes having no corresponding animal isolates of the same type, and resulted in greater uncertainty in the source attribution estimates. The level of uncertainty shown in Figure 1 is that derived from the number of samples contributing to the estimates, but there is additional uncertainty in that some subtyping approaches may be grouping together isolates that are not genuinely related. This could lead to misclassification of isolates leading to incorrect source attribution. Such uncertainty is problematic to quantify, and the optimal genetic distance over which to group together isolates would be a useful area of further research. One possible way to investigate this would be to apply source attribution approaches to outbreak data, where tracings have been undertaken and the sources of infection are known, at least for a proportion of the human cases. This would allow for comparison of the model attribution estimates with the observed data for a range of different thresholds, and could also allow comparison of different source attribution approaches.

The difficulty of choosing the appropriate level of discriminatory power of subtyping approaches has been previously discussed in de Knegt et al. (2016), where a Multiple Locus Variable Number Tandem Repeat Analysis (MLVA) approach was used to type SE and ST samples in Denmark. In this earlier study it was pointed out that at least the use of such typing approaches allows the model fit using different levels of discriminatory power to be compared, and for one to choose the one with the best fit. Using model fit as the criterion in the present study would suggest the use of the more discriminatory methods (Table 4). The use of MLVA has also been used for source attribution in Italy (Barco et al., 2015), this study using the asymmetric island approach to estimate source attribution for ST/MST, and finding a similar proportion of ST/MST attributed to pigs in Italy as the present study found for GB.

A key uncertainty in the model is the source of the human subtypes that occurred in humans alone. Even the subtyping approaches (e.g., 7-loci MLST or HCC100) that allowed for a relatively diverse set of isolates to be grouped together still had human isolates with no corresponding animal match. It is unclear whether these subtypes that occur in humans alone are from sources other than the animal ones considered in the present study or whether they are from less frequent types that occur in an animal source but was not in the subset of animal samples that were sequenced, which for most species was a very low number in this study. In practice, obtaining a representative set of animal samples that allows for comprehensive representation of the subtypes will be difficult as the level of surveillance varies between farm animal species, and even where National Control Programmes are carried out e.g., for *Salmonella* in poultry, sensitivity of the sampling methods is not high (Arnold et al., 2014). It should also be noted, that until sequencing is completed on all animal isolates, there is a risk that the sequenced population will be biased and more reflective of those related to human or animal outbreaks or those sequenced due to their unusual nature (e.g., antimicrobial resistance pattern). In fact, routine sequencing of all human isolates has taken place at PHE since April 2014, but currently sequencing is used for targeted investigations in animals.

There was considerable uncertainty in the estimation of the animal prevalence for each animal source. The source attribution and virulence factors appeared to be robust to the priors for the animal prevalence; in addition to the beta(1,1) and informative priors for prevalence, beta(0.5, 0.5) priors were also tried and made insignificant difference to the estimates (results not shown). This suggests that the key requirement for the estimation of these from sequenced animal isolates is a representative sample that covers the major subtypes rather than an accurate estimation of animal prevalence. However, the food source factors were sensitive to the priors for animal prevalence. Source attribution was the primary interest for the present study, but in other contexts there could be interest in the food source factors themselves, for example in ascertaining whether any particular sources are inherently more likely to be a vehicle for *Salmonella* transmission to humans than others, especially if one could also take into account the level of human exposure e.g., levels of consumption for foodborne sources. There was particular uncertainty in the estimates of prevalence for cattle and sheep, where passive surveillance data needed to be relied upon (e.g., APHA, 2016). Also, there are few data sources with which to estimate the prevalence for game birds and other mammals. For other mammals and game birds there are some data sources which can give an idea of the level of *Salmonella* occurrence. For example, for cats and dogs, there has been recording of the number of positive submissions for *Salmonella* through the SAVSNET scheme (Arsevska et al., 2017), and for horses and game birds there is similarly diagnostic submission data to APHA (APHA, 2014, 2015, 2016) but it is difficult to translate the number of diagnostic submissions for these species into actual animal infection prevalence.

The other mammals category was found to be an important source of human exposure to ST/MST in this study. It is likely that the major contributor to this is horses. In terms of confirmed laboratory diagnoses of ST/MST from livestock, horses had the second highest number after pigs between 2014 and 2016 (APHA, 2014, 2015, 2016). Other contributors to humans from the other mammals category are cats and dogs, but in a recent study these were found to have a low occurrence of ST/MST, with less than 1% of gastroenteritis submissions resulting in a positive test for *Salmonella*, and only a minority of those were ST/MST (Arsevska et al., 2017). The accuracy of the prediction of the source of human exposure using the Bayesian approach may increase with re-defining the model by increasing the number of isolates in each of the tested category and improving their representativeness of infections in the population.

## Conclusion

In conclusion, a Bayesian model has been piloted for source attribution using WGS data. This found that pigs were found to be the main contributor to human infection for ST/MST, followed by other mammals and cattle in the study period, although there is uncertainty in these estimates due to difficulties in robustly characterizing the subtype distribution for animal sources with low prevalence of these serovars or were less likely to have isolates selected for sequencing. It was found that the use of different clustering methods based on sequence data had minimal impact on the overall findings of the study, although the genetic distance over which isolates were grouped had an impact on the uncertainty estimates.

## Data Availability Statement

The datasets presented in this study can be found in online repositories. The names of the repository/repositories and accession number(s) can be found below: https://www.ebi.ac.uk/ena, PRJEB14853.

## Author Contributions

MA performed the data analysis. YT and JG generated the MLST, CgMLST, and SNP distance data in a format that could be used for the Bayesian modelling. RS and LP provided the oversight and input into the interpretation of the data and results. MA, RS, LP, and JG wrote the manuscript. All authors contributed to the article and approved the submitted version.

## Conflict of Interest

The authors declare that the research was conducted in the absence of any commercial or financial relationships that could be construed as a potential conflict of interest.

## Footnotes

1 https://www.compare-europe.eu/about

2 https://www.ebi.ac.uk/ena/data/view/PRJNA248792

3 https://www.computerome.dk

4 https://jgi.doe.gov/data-and-tools/bbtools/bb-tools-user-guide/bbduk-guide/

5 http://www.bioinformatics.babraham.ac.uk/projects/fastqc/

6 https://enterobase.warwick.ac.uk/species/index/senterica

7 https://enterobase.readthedocs.io/en/latest/features/clustering.html

## References

[B1] Achtman M. Wain J. Weill F. X. Nair S. Zhou Z. Sangal V. (2012). Multilocus sequence typing as a replacement for serotyping in *Salmonella enterica*. *PLoS Pathog.* 8:e1002776. 10.1371/journal.ppat.1009040 22737074 PMC3380943

[B2] Alikhan N. F. Zhou Z. Sergeant M. J. Achtman M. (2018). A genomic overview of the population structure of *Salmonella*. *PLoS Genet* 14:e1007261. 10.1371/journal.pgen.1007261 29621240 PMC5886390

[B3] APHA (2014). *Salmonella In Livestock Production.* United Kingdom: Animal and Plant Health Agency.

[B4] APHA (2015). *Salmonella In Livestock Production.* United Kingdom: Animal and Plant Health Agency.

[B5] APHA (2016). *Salmonella in Livestock Production.* United Kingdom: Animal and Plant Health Agency.

[B6] Arnold M. E. Gosling R. J. Martelli F. Mueller-Doblies D. Davies R. H. (2015). Evaluation of the sensitivity of faecal sampling for detection of monophasic *Salmonella* Typhimurium and other *Salmonella* in cattle and pigs. *Epidemiol. Infect.* 143 1681–1691. 10.1017/s0950268814002453 25266772 PMC9507225

[B7] Arnold M. E. Martelli F. Mclaren I. Davies R. H. (2014). Estimation of the sensitivity of environmental sampling for detection of *Salmonella* in commercial layer flocks post-introduction of national control programmes. *Epidemiol. Infect.* 142 1061–1069. 10.1017/s0950268813002173 24020913 PMC9151119

[B8] Arnold M. E. Mueller-Doblies D. Carrique-Mas J. J. Davies R. H. (2009). The estimation of pooled-sample sensitivity for detection of *Salmonella* in turkey flocks. *J. Appl. Microbiol.* 107 936–943. 10.1111/j.1365-2672.2009.04273.x 19320955

[B9] Arsevska E. Singleton D. Sanchez-Vizcaino F. Williams N. Jones P. H. Smyth S. (2017). Small animal disease surveillance: GI disease and salmonellosis. *Vet. Rec.* 181 228–232. 10.1136/vr.j3642 28864509

[B10] Bankevich A. Nurk S. Antipov D. Gurevich A. A. Dvorkin M. Kulikov A. S. (2012). SPAdes: a new genome assembly algorithm and its applications to single-cell sequencing. *J. Comput. Biol.* 19 455–477. 10.1089/cmb.2012.0021 22506599 PMC3342519

[B11] Barco L. Barrucci F. Cortini E. Ramon E. Olsen J. E. Luzzi I. (2015). Ascertaining the relationship between *Salmonella* Typhimurium and *Salmonella* 4,[5],12:i:- by MLVA and inferring the sources of human salmonellosis due to the two serovars in Italy. *Front. Microbiol.* 6:301. 10.3389/fmicb.2015.00301 25983720 PMC4415582

[B12] Dallman T. Ashton P. Schafer U. Jironkin A. Painset A. Shaaban S. (2018). SnapperDB: a database solution for routine sequencing analysis of bacterial isolates. *Bioinformatics* 34 3028–3029. 10.1093/bioinformatics/bty212 29659710

[B13] de Knegt L. V. Pires S. M. Lofstrom C. Sorensen G. Pedersen K. Torpdahl M. (2016). Application of Molecular Typing Results in Source Attribution Models: The Case of Multiple Locus Variable Number Tandem Repeat Analysis (MLVA) of *Salmonella* Isolates Obtained from Integrated Surveillance in Denmark. *Risk Anal.* 36 571–588. 10.1111/risa.12483 27002674

[B14] ECDC (2016). *Annual Epidemiological Report for 2016, Salmonellosis.* Sweden: ECDC.

[B15] EFSA (2012). The European Summary Report on Trends and Sources of Zoonoses, Zoonotic Agents and Food-Borne Outbreaks, 2010. *EFSA J.* 10 2597. 10.2903/j.efsa.2012.2597 PMC13151809 42111985

[B16] Food Control Consultants Ltd Consortium [FCC] (2011). *Analysis of the costs and benefits of setting a target for the reduction of Salmonella in slaughter pigs for European Commission Health and Consumers Directorate-General SANCO/2008/E2/036 Final Report. 2010:1–198.* England: FCC, 2010 1–198.

[B17] Gelman A. E. John B. C. Hal S. S. (2003). *Bayesian Data Analysis*, 2nd Edn Boca Raton: Chapman and Hall/CRC Press.

[B18] Glass K. Fearnley E. Hocking H. Raupach J. Veitch M. Ford L. (2016). Bayesian Source Attribution of Salmonellosis in South Australia. *Risk Anal.* 36 561–570. 10.1111/risa.12444 26133008

[B19] Guillier L. Gourmelon M. Lozach S. Cadel-Six S. Vignaud M. L. Munck N. (2020). AB_SA: Accessory genes-Based Source Attribution - tracing the source of *Salmonella enterica* Typhimurium environmental strains. *Microb. Genom.* 6:mgen000366. 10.1099/mgen.0.000366 PMC7478624 32320376

[B20] Gurevich A. Saveliev V. Vyahhi N. Tesler G. (2013). QUAST: quality assessment tool for genome assemblies. *Bioinformatics* 29 1072–1075. 10.1093/bioinformatics/btt086 23422339 PMC3624806

[B21] Hald T. Lo, Fo Wong D. M. Aarestrup F. M. (2007). The attribution of human infections with antimicrobial resistant *Salmonella* bacteria in Denmark to sources of animal origin. *Foodborne Pathog. Dis.* 4 313–326. 10.1089/fpd.2007.0002 17883315

[B22] Hald T. Vose D. Wegener H. C. Koupeev T. (2004). A Bayesian approach to quantify the contribution of animal-food sources to human salmonellosis. *Risk Anal.* 24 255–269. 10.1111/j.0272-4332.2004.00427.x 15028016

[B23] Lupolova N. Dallman T. J. Holden N. J. Gally D. L. (2017). Patchy promiscuity: machine learning applied to predict the host specificity of *Salmonella enterica* and *Escherichia coli*. *Microb. Genom.* 3:e000135. 10.1099/mgen.0.000135 PMC5695212 29177093

[B24] Merlotti A. Manfreda G. Munck N. Hald T. Litrup E. Nielson E. M. (2020). Network Approach to Source Attribution of *Salmonella enterica* Serovar Typhimurium and Its Monophasic Variant. *Front. Microbiol.* 11:1205 10.3389/fmicb.2020.01205 PMC8335978 34354676

[B25] Milnes A. S. Stewart I. Clifton-Hadley F. A. Davies R. H. Newell D. G. Sayers A. R. (2008). Intestinal carriage of verocytotoxigenic *Escherichia coli* O157, *Salmonella*, thermophilic Campylobacter and Yersinia enterocolitica, in cattle, sheep and pigs at slaughter in Great Britain during 2003. *Epidemiol. Infect.* 136 739–751. 10.1017/s0950268807009223 17655782 PMC2870870

[B26] Mueller-Doblies D. Speed K. C. R. Kidd S. Davies R. H. (2018). *Salmonella* Typhimurium in livestock in Great Britain - trends observed over a 32-year period. *Epidemiol. Infect.* 146 409–422. 10.1017/s095026881800002x 29415790 PMC9134571

[B27] Mughini-Gras L. Barrucci F. Smid J. H. Graziani C. Luzzi I. Ricci A. (2014). Attribution of human *Salmonella* infections to animal and food sources in Italy (2002-2010): adaptations of the Dutch and modified Hald source attribution models. *Epidemiol. Infect.* 142 1070–1082. 10.1017/s0950268813001829 23920400 PMC9151150

[B28] Mullner P. Jones G. Noble A. Spencer S. E. Hathaway S. French N. P. (2009). Source attribution of food-borne zoonoses in New Zealand: a modified Hald model. *Risk Anal.* 29 970–984. 10.1111/j.1539-6924.2009.01224.x 19486473

[B29] Munck N. Leekitcharoenphon P. Litrup E. Kaas R. Meinen A. Guillier L. (2020a). Four European *Salmonella* Typhimurium datasets collected to develop WGS-based source attribution methods. *Sci. Data* 7:75. 10.1038/s41597-020-0417-7 PMC7054362 32127544

[B30] Munck N. Njage P. M. N. Leekitcharoenphon P. Litrup E. Hald T. (2020b). Application of Whole-Genome Sequences and Machine Learning in Source Attribution of *Salmonella* Typhimurium. *Risk Anal.* 40 1693–1705. 10.1111/risa.13510 32515055 PMC7540586

[B31] Nerette P. Stryhn H. Dohoo I. Hammell L. (2008). Using pseudogold standards and latent-class analysis in combination to evaluate the accuracy of three diagnostic tests. *Prev. Vet. Med.* 85 207–225. 10.1016/j.prevetmed.2008.01.011 18355935

[B32] Petrovska L. Mather A. E. Abuoun M. Branchu P. Harris S. R. Connor T. (2016). Microevolution of Monophasic *Salmonella* Typhimurium during Epidemic, United Kingdom, 2005-2010. *Emerg. Infect. Dis.* 22 617–624. 10.3201/eid2204.150531 26982594 PMC4806966

[B33] PHE (2018). *Salmonella data 2007 to 2016.* England: PHE.

[B34] Powell L. F. Cheney T. E. Williamson S. Guy E. Smith R. P. Davies R. H. (2016). A prevalence study of *Salmonella* spp., Yersinia spp., Toxoplasma gondii and porcine reproductive and respiratory syndrome virus in UK pigs at slaughter. *Epidemiol. Infect.* 144 1538–1549. 10.1017/s0950268815002794 26586451 PMC9150582

[B35] Tam C. C. O’brien S. J. Tompkins D. S. Bolton F. J. Berry L. Dodds J. (2012a). Changes in causes of acute gastroenteritis in the United Kingdom over 15 years: microbiologic findings from 2 prospective, population-based studies of infectious intestinal disease. *Clin. Infect. Dis.* 54 1275–1286. 10.1093/cid/cis028 22412058

[B36] Tam C. C. Rodrigues L. C. Viviani L. Dodds J. P. Evans M. R. Hunter P. R. (2012b). Longitudinal study of infectious intestinal disease in the UK (IID2 study): incidence in the community and presenting to general practice. *Gut* 61 69–77. 10.1136/gut.2011.238386 21708822 PMC3230829

[B37] Tewolde R. Dallman T. Schaefer U. Sheppard C. L. Ashton P. Pichon B. (2016). MOST: A modified MLST typing tool based on short read sequencing. *PeerJ.* 4:e2308. 10.7717/peerj.2308 27602279 PMC4991843

[B38] Zhang S. Yin Y. Jones M. B. Zhang Z. Deatherage Kaiser B. L. Dinsmore B. A. (2015). *Salmonella* serotype determination utilizing high-throughput genome sequencing data. *J. Clin. Microbiol.* 53 1685–1692. 10.1128/jcm.00323-15 25762776 PMC4400759

