# Supplementary material for: Bayesian Source Attribution of Salmonella Typhimurium Isolates From Human Patients and Farm Animals in England and Wales
Source: Front Microbiol. 2021 Jan 28;12:579888. doi: 10.3389/fmicb.2021.579888 (PMC7876086; doi:10.3389/fmicb.2021.579888)
Supplement: Supplementary file 1 [file Data_Sheet_2.docx]

Supplementary Material

# Supplementary Table

**Supplementary Table S1**

Distribution of *S*. Typhimurium (STM) and monophasic variant isolates included in a Bayesian source attribution study for England and Wales, grouped by animal/human source.

| Source | STM | 4,12:i:- | 4,5,12:i:- | Total |
| --- | --- | --- | --- | --- |
| Broilers | 4 | 2 | 3 | 9 |
| Layers | 2 | 4 | 1 | 7 |
| Turkey | 5 | 0 | 9 | 14 |
| Game birds^1^ | 18 | 0 | 0 | 18 |
| Cattle | 7 | 6 | 7 | 20 |
| Pigs | 40 | 35 | 88 | 163 |
| Sheep | 6 | 0 | 1 | 7 |
| Other mammals^2^ | 27 | 4 | 11 | 42 |
| Total animal | 109 | 51 | 120 | 280 |
| Human | 83 | 17 | 77 | 177 |

1. - For the 18 game bird isolates there were: 6 from pheasants, 4 from pigeon, 8 from quails
2. - For the 42 other mammals isolates there were: 3 from cats, 17 from dog, 22 from horses

# Supplementary Figure

## Supplementary Figures


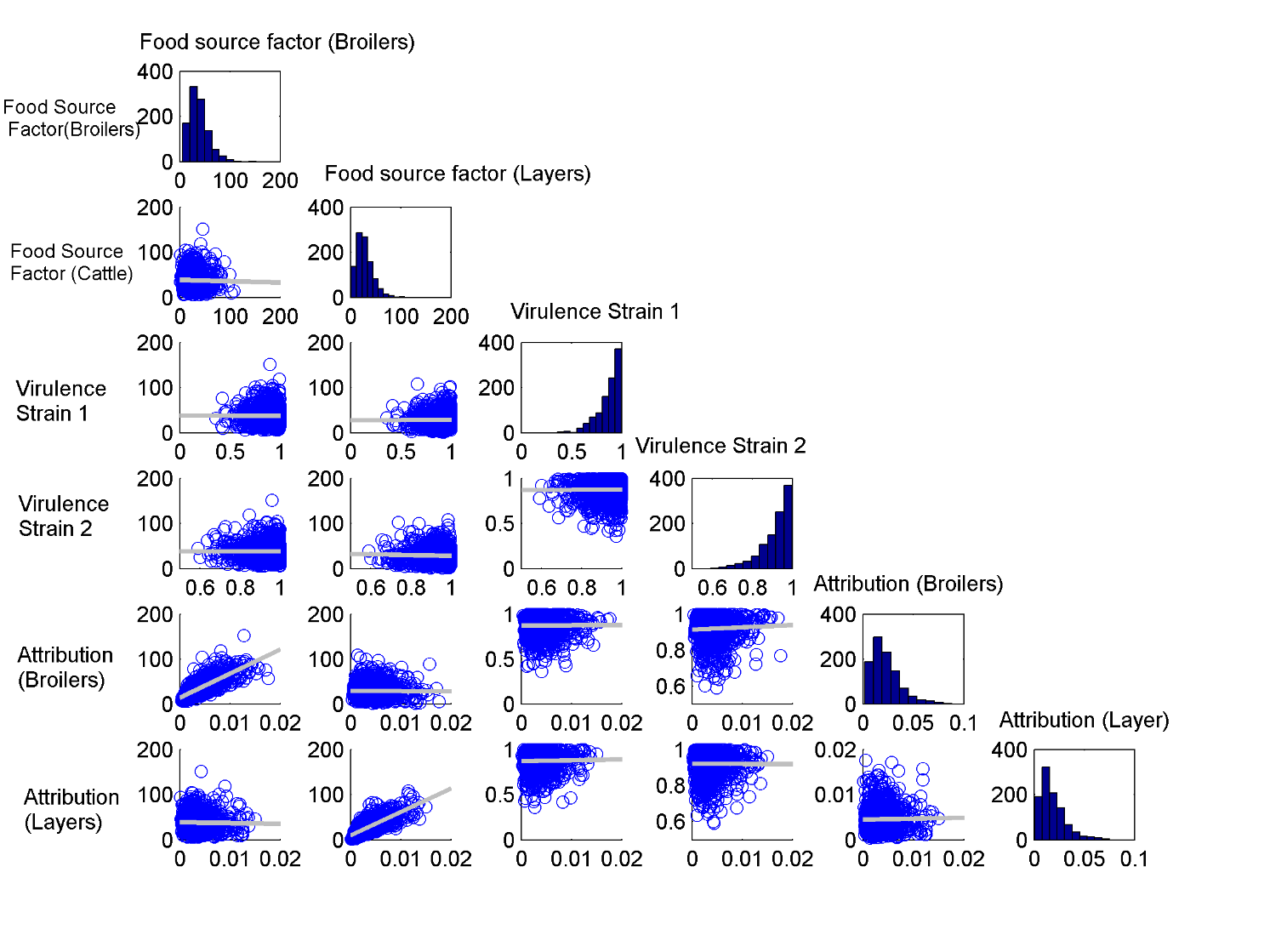


**Supplementary Figure 1.** Correlation matrix plot between a selection of the parameters for a Bayesian source attribution model for Salmonella, using the SNP10 subtyping method. Correlation between parameters is low between the majority of parameters, except for between the food source factor and source attribution estimates for broilers and layers. The full matrix of parameter correlation estimates for the SNP10 subtyping method is given in a spreadsheet as supplementary data (CorrCoeffsSNP10.xls).
